# Withaferin A Inhibits the Proteasome Activity in Mesothelioma *In Vitro* and *In Vivo*

**DOI:** 10.1371/journal.pone.0041214

**Published:** 2012-08-17

**Authors:** Huanjie Yang, Ying Wang, Vino T. Cheryan, Wenjuan Wu, Cindy Qiuzhi Cui, Lisa A. Polin, Harvey I. Pass, Q. Ping Dou, Arun K. Rishi, Anil Wali

**Affiliations:** 1 John D. Dingell VA Medical Center, Wayne State University, Detroit, Michigan, United States of America; 2 Karmanos Cancer Institute, Wayne State University, Detroit, Michigan, United States of America; 3 Departments of Oncology, Wayne State University, Detroit, Michigan, United States of America; 4 Department of Pathology, Wayne State University, Detroit, Michigan, United States of America; 5 New York University Cancer Center, New York, New York, United States of America; 6 Department of Life Science and Engineering, Harbin Institute of Technology, Harbin, China; University of Kansas Medical Center, United States of America

## Abstract

The medicinal plant *Withania somnifera* has been used for over centuries in Indian Ayurvedic Medicine to treat a wide spectrum of disorders. Withaferin A (WA), a bioactive compound that is isolated from this plant, has anti-inflammatory, immuno-modulatory, anti-angiogenic, and anti-cancer properties. Here we investigated malignant pleural mesothelioma (MPM) suppressive effects of WA and the molecular mechanisms involved. WA inhibited growth of the murine as well as patient-derived MPM cells in part by decreasing the chymotryptic activity of the proteasome that resulted in increased levels of ubiquitinated proteins and pro-apoptotic proteasome target proteins (p21, Bax, IκBα). WA suppression of MPM growth also involved elevated apoptosis as evidenced by activation of pro-apoptotic p38 stress activated protein kinase (SAPK) and caspase-3, elevated levels of pro-apoptotic Bax protein and cleavage of poly-(ADP-ribose)-polymerase (PARP). Our studies including gene-array based analyses further revealed that WA suppressed a number of cell growth and metastasis-promoting genes including c-myc. WA treatments also stimulated expression of the cell cycle and apoptosis regulatory protein (CARP)-1/CCAR1, a novel transducer of cell growth signaling. Knock-down of CARP-1, on the other hand, interfered with MPM growth inhibitory effects of WA. Intra-peritoneal administration of 5 mg/kg WA daily inhibited growth of murine MPM cell-derived tumors *in vivo* in part by inhibiting proteasome activity and stimulating apoptosis. Together our *in vitro* and *in vivo* studies suggest that WA suppresses MPM growth by targeting multiple pathways that include blockage of proteasome activity and stimulation of apoptosis, and thus holds promise as an anti-MPM agent.

## Introduction

Malignant pleural mesothelioma (MPM) is a lethal asbestos-related malignancy [1]. Despite aggressive multimodality treatment involving surgery, adjuvant or neoadjuvant chemotherapy, and radiation [2], the median survival of MPM is about 9–17 months [3]. Millions of American workers have been exposed to asbestos, and exposure to asbestos has been shown to increase the risk of several serious diseases including asbestosis, lung cancer and mesothelioma [1]. It is estimated that there are 2,000 to 3,000 people diagnosed as MPM patients each year in the United States and the incidence of this disease is expected to increase in the next decade in United States and Europe [3], [4]. Due to the resistance to currently available chemotherapies and the increasing incidence of MPM, development of new treatments for MPM is urgently needed.

A number of studies suggest that agents derived from plants including dietary fruits and vegetables are helpful in either inhibiting or reversing the development of cancer [5]–[7]. A medicinal plant, *Withania somnifera*, is commonly used in Indian traditional medicine to treat a range of disorders [8]. Withaferin-A (WA), a steroidal lectone is a bioactive compound isolated from Withania somnifera, exhibits anti-inflammatory, immunomodulatory, and anti-angiogenic properties [9], [10]. WA suppresses growth of human cancer cells of prostate, breast, and soft tissue sarcoma origins *in vitro* and *in vivo* [11]–[13]. Although the mechanisms of action of WA have yet to be fully defined, the pro-survival NF-κB and AKT, pro-apoptotic Par-4, FOXO-3, and Bim, and the proteasomal chymotrypsin subunit β5 have been shown to be molecular targets of WA signaling [11], [14]–[16]. WA treatments cause alterations in the cytoskeletal architecture, induce generation of reactive oxygen species, mitochondrial dysfunction and proteasomal inhibition [14], [17], [18].

Here we investigated effects of WA on growth of MPM cells *in vitro*. Consistent with observations in other models, WA caused cell growth inhibition that involved diminished proteasomal activities and elevated apoptosis. Although WA stimulated expression of pro-apoptotic Bax, and activated p38 SAPK and caspase-3/7, our gene-array-based analysis revealed that WA suppressed expression of a number of cell growth and metastasis transducers. Moreover, WA suppression of MPM cell growth involved stimulation of a novel transducer of cell growth and apoptosis signaling CARP-1/CCAR1 [19]–[21]. Intra-peritoneal administration of WA suppressed growth of murine mesothelioma allografts in part by enhancing apoptosis. Our proof-of-concept studies reveal, for the first time, MPM inhibitory properties of WA and are expected to facilitate utilization of this agent or its potent derivatives as potential adjuvants for treatment and perhaps chemoprevention of MPM.

## Methods

### Cells and Reagents

MPM patient derived cell lines [H2373, H2452, H2461, and H2595] established in our laboratory and characterized in detail [22] were cultured in RPMI 1640 supplemented with 100 units/ml of penicillin, 100 µg/ml streptomycin, 4 mM L-glutamine, and 10% fetal calf serum. H226 MPM cells were obtained from ATCC (Manassas, VA) and maintained following vendor's guidelines. The AB12 murine malignant mesothelioma cell line was derived from BALB/c mice and was shown to form subcutaneous tumors when implanted in mice [23], [24]. This cell line was cultured and maintained in high-glucose DMEM supplemented with 10% fetal bovine serum (FBS), 100 units/mL penicillin, 100 µg/mL streptomycin. FBS was obtained from Tissue Culture Biologicals (Tulare, CA). RPMI 1640 medium, penicillin and streptomycin were purchased from Invitrogen Co. (Carlsbad, CA). DMEM was purchased from Mediatech Inc (Herndon, VA). Cells were incubated at 37°C in a humidified atmosphere of 5% CO_2_ in air and were passaged weekly. Purified withaferin A (>98%) was purchased from ChromaDex, Inc. (Santa Ana, CA) and dissolved in dimethyl sulfoxide (DMSO; Sigma; St. Louis, MO) at a stock concentration of 50 mM, aliquoted and stored at −20°C. Purified rabbit *20S* proteasome, mouse monoclonal antibody p21, fluorogenic substrates N-Succinyl-Leu-Leu-Val-Tyr-7-amino-4-methylcoumarin (Suc-LLVY-AMC) for the proteasomal chymotryptic activity and the caspase-3/-7-specific substrate N-acetyl-Asp-Glu-Val-Asp-7-amino-4-methylcoumarin (Ac-DEVD-AMC) were obtained from Calbiochem Inc. (San Diego, CA). Anti-PARP mouse monoclonal antibody was purchased from BIOMOL International LP (Plymouth Meeting, PA). Anti-Bax (B-9), anti-p27 (F-8), anti-c-myc (9E10), and anti-Ubiquitn (P4D1) mouse monoclonal antibodies as well as anti-inhibitor of nuclear factor κB-α (IκB-α) (C-15), anti-c-Jun (H-79), anti-vimentin (V9) rabbit polyclonal, and anti-actin (C-11) goat polyclonal antibodies were obtained from Santa Cruz Biotechnology Inc. (Santa Cruz, CA). Mouse monoclonal antibody NCL-p27 was purchased from Novocastra Laboratories Ltd (Newcastle upon Tyne, UK). Anti-p38 and phospho-p38 rabbit polyclonal antibodies were obtained from Cell Signaling (Beverly, MA). Generation and characterization of the anti-CARP-1/CCAR1 rabbit polyclonal antibodies have been described before [19]. Enhanced Chemiluminescence Reagent was purchased from Amersham Biosciences (Piscataway, NJ) and the Apoptag Peroxidase in situ Apoptosis Detection Kit was obtained from Chemicon International, Inc. (Temecula, CA). Protein Assay Kit was purchased from Bio-Rad Laboratories (Hercules, CA), while 3–4, 5-dimethyltiazol-2-yl-2.5-diphenyl-tetrazolium bromide (MTT), cremophor and other chemicals were obtained from Sigma-Aldrich (St. Louis, MO). The ON-Target plus SiRNAs for knock-down of CARP-1 and DharmaFECT transfection reagent for Si-RNA transfections were purchased from Dharmacon Inc., Thermo Fisher Scientific (Lafayette, CO).

### Cell Growth Inhibition Studies by MTT Assay

MPM (H2373, H2452, H2461, H226 and AB12) cells (5×10^3^) were seeded in a 96-well culture plate and subsequently treated with WA at different concentrations for noted times. Control cells were treated with 0.1% DMSO in culture medium. After treatment, the cells were incubated with 1 mg/ml of MTT reagent at 37°C for 4 h and then MTT was removed and 100 µL of DMSO was added, followed by colorimetric analysis using a multilabel plate reader at 560 nm (Victor^3^; PerkinElmer, Wellesley, MA, USA).

### Inhibition of cellular 26S proteasome activity

MPM cells were treated with either DMSO or WA for indicated times, followed by extraction of whole cell lysate. Proteins from whole cell lysate were incubated with the proteasomal chymotrypsin-like specific substrate Suc-LLVY-AMC (at 20 µM). The proteasomal activity was measured by hydrolysis of their substrates, with 355-nm excitation and 460-nm emission wavelengths.

### Cell-free Caspase-3/-7 activity assay

MPM cells were treated with different concentrations of WA for indicated time periods. The prepared whole cell extract (30 µg per sample) was then incubated with 40 µM of caspase-3/-7 substrate Ac-DEVD-AMC in 100 µl of the assay buffer (20 mM Tris–HCl, pH 7.5) at 37°C for at least 2 h. The release of the AMC groups was measured as above.

### Western blot analysis

Cells were harvested and lysed in RIPA buffer (50 mM Tris-HCI, pH 8.0, 150 mM sodium chloride, 1.0% NP-40, 0.5% sodium deoxycholate, 0.1% sodium dodecyl sulfate (SDS), and 0.1% of protease inhibitor cocktail) for 20 min at 4°C. The lysates were centrifuged at 14,000 rpm at 4°C for 15 min to remove debris. Protein concentrations of whole cell lysates were determined using the Protein Assay Kit. Supernatant proteins, 50 µg from each sample, were separated by SDS-10% polyacrylamide gel electrophoresis (SDS-PAGE) and transferred to polyvinylidene difluoride (PVDF) membrane (Bio-rad, Hercules, CA) by standard procedures. The membranes were hybridized with primary antibodies followed by incubation with appropriate secondary antibodies. The antibody-bound proteins were visualized by treatment with the chemiluminescence detection reagent (Pierce) according to manufacturer's instructions, followed by exposure to film (Kodak X-Omat). The same membrane was re-probed with the anti-β actin antibody, which was used as an internal control for protein loading.

### Isolation of RNA and microarray analysis

Total RNA was extracted from untreated or WA-treated H2373 MPM cells. At the end of treatments, the untreated and treated cells were harvested and total RNA were isolated, and purified using the RNeasy Mini kit and RNase-free DNase Set (Qiagen, Valencia, CA) according to the manufacturer's protocols. WA-dependent changes in gene expression in MPM cells were performed at the Genomic Core Facility, Karmanos Cancer Institute utilizing Illumina BeadChip® Arrays essentially according to manufacturer's instruction (Illumina). Briefly, 0.5 µg total RNA was biotin-labeled and hybridized with BeadChips. The signal was detected with streptovadin-Cy3 according to manufacturer's instruction (Illumina). The imaging of the BeadChips was conducted using a Bead Array Reader in conjunction with Bead Studio software (Illumina). Normalization of the data was carried out using a quantile based approach which transforms the raw data so that the resulting normalized expression values of each sample have the same distribution [25]. An unsupervised cluster analysis was performed to detect similarities among samples based on gene expression profiles. The genes retained to perform the clustering were those varying the most regardless their group membership as described elsewhere [26]. Significance of the differentially expressed genes among various groups was tested using a moderated *t*-test to allow for *p*-value computation for the significance of gene changes. The *p*-values were then adjusted using the False Discovery Rate method [26] to derive corrected *p*-values. The p values of <0.5 were considered significant provided that the fold change in expression was also equal to or larger than 2.

### Murine mesothelioma allograft experiments

All animal work was done in accordance with protocols approved by the Institutional Laboratory Animal Care and Use Committee of Wayne State University. Female BALB/c mice aged 5 weeks were purchased from Taconic Research Animal Services and housed in accordance with protocols approved by the WSU IACUC. On day 1 AB12 murine mesothelioma cells (0.5×10^6^) were suspended in 0.1 mL serum-free DMEM medium and inoculated subcutaneously (*s.c.*) in the right flank of each mouse (n = 5 each group). When the tumors became palpable (on day 10 after inoculation), the mice were randomly assigned into 2 groups. The control group animals received a daily *i.p.* injection with 50–100 µl of a vehicle [10% DMSO, 70% Cremophor∶ethanol (3∶1) and 20% PBS] while the test group animals received 50–100 µl of 5.0 mg/kg WA daily. Tumor sizes were measured daily using calipers and their volumes calculated using a standard formula: width^2^×length/2. Body weight was measured weekly. The mice were sacrificed after 16 day-treatment when control tumors reached to ∼1774 mm^3^. H&E staining confirmed the presence of tumor.

### Immunohistochemical analysis

Apoptosis in tumor tissues was determined by TUNEL assay using in situ cell Death Detection kit from Roche Applied Science (Indianapolis, IN) according to the manufacturer's instruction. The formalin-fixed tumor xenograft biopsies from untreated or WA-treated animals were paraffin embedded and processed essentially following our previously described procedures [27]. The tumor tissue slides were stained for presence of CARP-1, cyclin-dependent kinase inhibitor p27, and oncogene c-myc by utilizing respective antibodies, and were then photographed under different magnifications using Zeiss microscope with a 35 mm camera attached for recording the photomicrographs. H&E counter-staining of tumor tissues was performed following our previously described methods [27]. The proteasomal or caspase activity assays and Western blotting using animal tumor tissue samples were performed similarly as described above using cultured MPM cells.

## Results

### WA inhibits MPM cell proliferation

**Figure 1:**
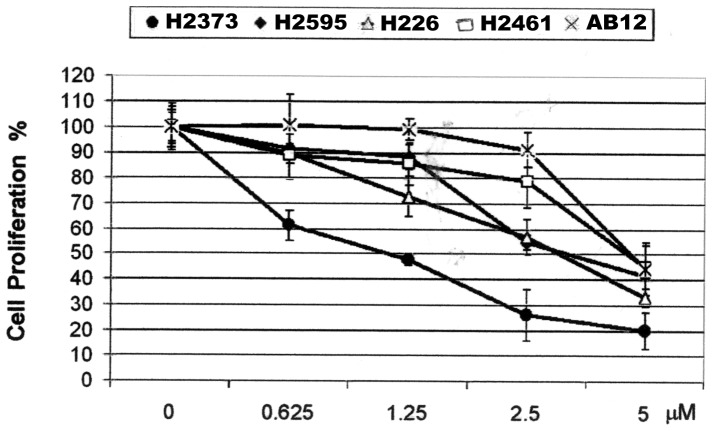
Antiproliferative effect of WA on human MPM cells. Cells were treated with vehicle (Control, denoted as 0) or indicated doses of WA for 72 h. Determination of viable/live cells was carried out by MTT assay. The data in the histograms represent means of three independent experiments; bars, S.E.

We first tested the growth-inhibitory effects of WA on four human MPM cell lines that were derived from mesothelioma patients and one murine mesothelioma cell line. WA potently inhibited cell proliferation in all tested cell lines (Fig. 1). Among them, H2373 cells were most sensitive towards WA treatment, showing 52% inhibition of their growth in the presence of 1.25 µM dose (Fig. 1). H2595, H226, H2452 and AB12 cell lines were relatively less sensitive to WA treatment, exhibiting 58, 67, 55 and 55% inhibition, respectively, at 5 µM dose (Fig. 1).

### WA inhibits proteasome activity and stimulates apoptosis in MPM cells

**Figure 2:**
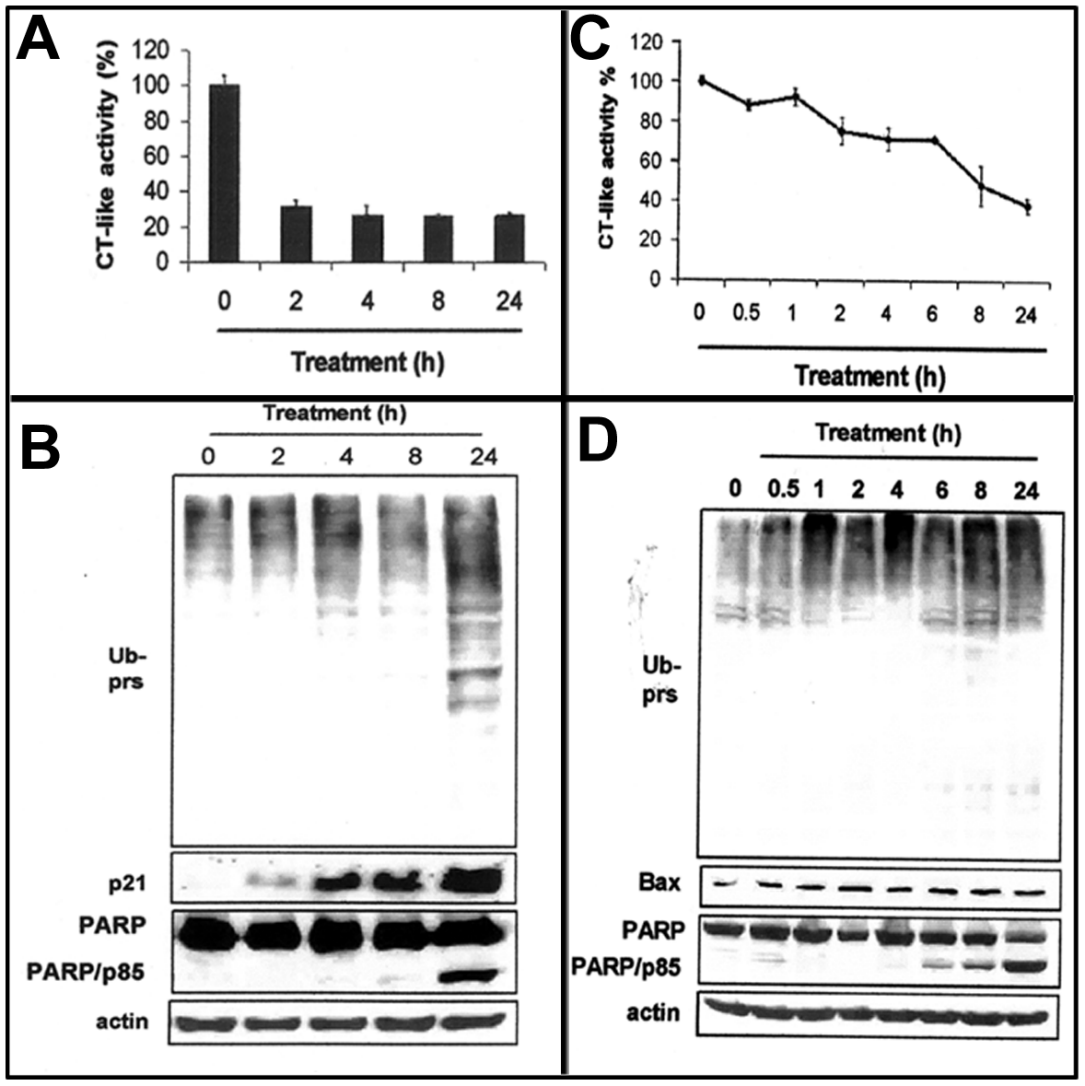
WA inhibits MPM cell proteasome activity. (A, B) Kinetic effects of WA on proteasome inhibition and apoptosis induction in H2595 human MPM cells. Cells were treated with 10 µM WA for various times. Chymotryptic activity of the proteasome was determined as in methods (A) and accumulation of ubiquitinated proteins, levels of p21, PARP, cleaved PARP, and actin proteins were determined by Western blotting (B). (C, D) WA inhibits proteasomal chymotrypsin-like activity in AB12 murine MPM cells and induces apoptosis. Cells were treated with 10 µM WA for indicated time periods, followed by measurement of proteasomal CT-like activity (C), Western blotting analysis for accumulation of ubiquitinated proteins, and levels of Bax, PARP, cleaved PARP, and actin proteins (D) as described in methods. *Bars*, SD; Ub prs, Ubiquitinated proteins.

WA was previously reported to target multiple molecules, such as FOX3a, or the proteasome [11], [14], to suppress cell growth. Consistent with these observations, treatments of H2595 MPM cells with 10 µM dose of WA for up to 24 h elicited about 70% inhibition of the proteasomal chymotrypsin-like activity (Fig. 2A). The proteasome inhibition was noted as early as 2 h of WA exposure, and remained inhibited to the same extent for the rest of the treatment periods (Fig. 2A). Similar to human MPM cells, WA also inhibited the proteasomal chymotrypsin-like activity in murine AB12 MPM cells (Fig. 2C). The chymotryptic activity of the proteasome was inhibited around 10% within 1 h after addition of 5 µM WA and further decreased by 25–30% during 2–6 h and leading to 52 to 62% decline in proteasomal activity over 8–24 h (Fig. 2C). Western blot analysis that showed an increased level of ubiquitinated proteins after 2–24 h of WA treatment in both the human and murine MPM cells (Fig. 2B, D), as well as the accumulation of the proteasomal targets protein p21 and Bax proteins (Fig. 2B, D) suggesting inhibition of proteasome by WA.

**Figure 3:**
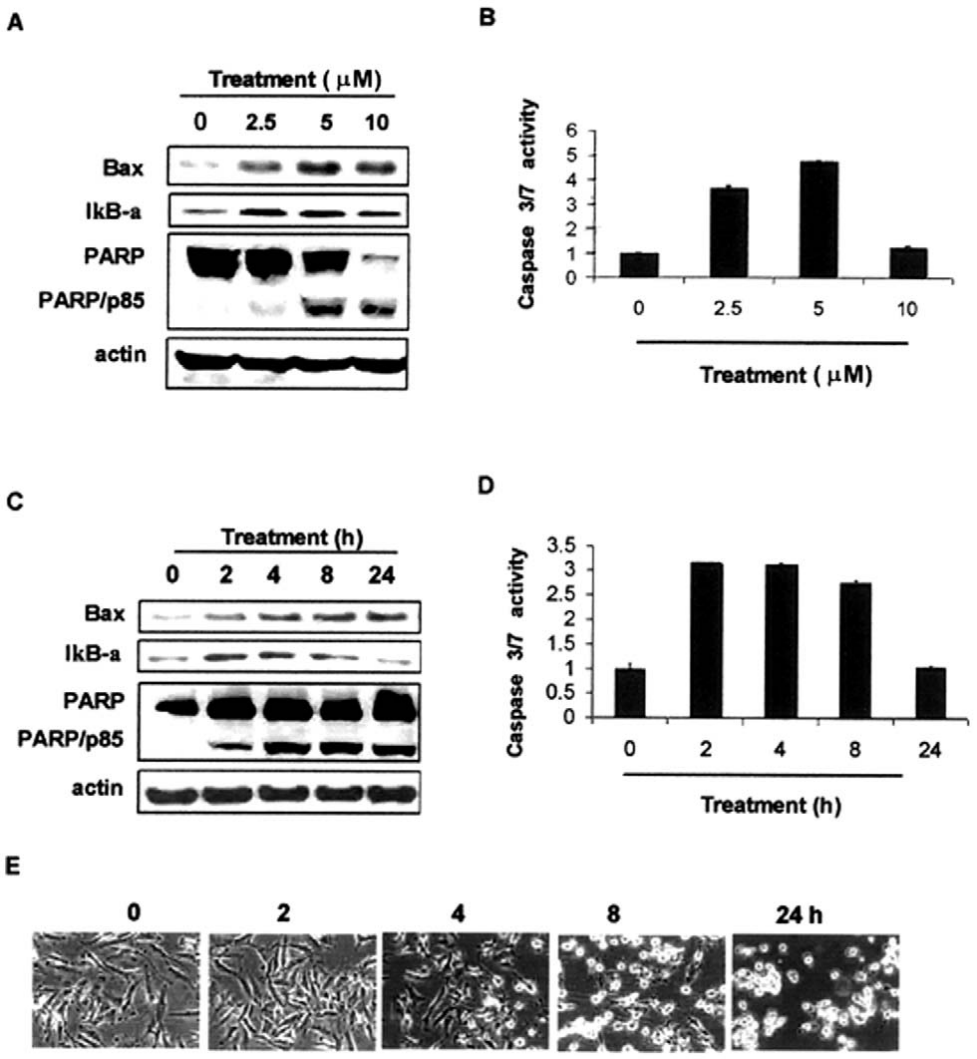
Dosage and kinetic effects of WA on apoptosis induction in human MPM cells. H2373 cells were treated with different concentrations of WA for 16 h (A, B), or 10 µM WA for various times (C–E), followed by Western blotting analysis for levels of Bax, IκB-α, PARP, and actin proteins as detailed in methods (A and C). B, D, Caspase 3/7 activation was determined from by WA treated cell lysates as in methods. *Bars*, SD. E, Photomicrographs showing apoptosis-associated morphological changes in WA-treated MPM cells for indicated times. 0, Control, DMSO treatment.

Suppression of anti-apoptotic/pro-survival signaling pathway and/or activation of apoptotic pathway are one of the mechanisms that contribute to cell growth inhibition. WA treatments caused a time-dependent increase in levels of cell cycle inhibitory protein p21, pro-apoptotic protein Bax, and IκB-α, an endogenous inhibitor of anti-apoptotic NF-κB (Figs. 2B, D, and 3A, C), suggesting that WA inhibited MPM cells growth in part by causing apoptosis. Indeed, apoptosis induction was confirmed by multiple assays. First, WA treatments caused increased cleavage of PARP in MPM cells. As shown in Fig. 3A, a moderate PARP cleavage was observed in cells treated with 2.5 µM WA, while a robust cleavage of PARP was noticeable in MPM cells treated with 5–10 µM WA (Fig. 3A). Second, a 3.6–4.7-fold increase in caspase 3/7 activity was detected in 2.5–5 µM WA treated H2373 cells (Fig. 3B). WA treatments also caused caspase 3/7 activation in a time-dependent manner in murine MPM cells (not shown). Finally, morphological changes associated with apoptotic nuclei were observed in 10 µM WA treated cells (data not shown, but see Fig. 3E). We next studied kinetic effects of WA on apoptosis induction in H2373 cells. Levels of the pro-apoptotic protein Bax began to increase after 2 h of WA treatment and showed maximal increase at 24 h treatment (Fig. 3C). IκB-α levels however peaked after 2–4 h of WA treatment, and then dropped to the basal level at 24 h treatment (Fig. 3C). Increase in Bax protein in the presence of WA accompanied consistent ∼3-fold activation of caspase 3/7 and cleavage of PARP and apoptosis-associated morphological changes in MPM cells (Figs. 3C, D, and E).

Although, WA inhibited proteasomal activity in H2595 MPM cells within 2 h of treatment (Fig. 2A), the Western blotting experiments revealed cleavage of PARP at the 24 h treatment period (Fig. 2B). Moreover, WA treatment failed to activate caspase 3/7 in H2595 cells (data not shown), suggesting that caspase 3/7-independent cleavage of PARP likely contributed to relative resistance of the H2595 cells to growth inhibition by WA. Taken together, our *in vitro* studies strongly suggest that WA suppressed growth of human and murine MPM cells by attenuating proteasomal activity and stimulating apoptosis.

### WA suppression of MPM growth involves novel apoptosis transducer

**Figure 4:**
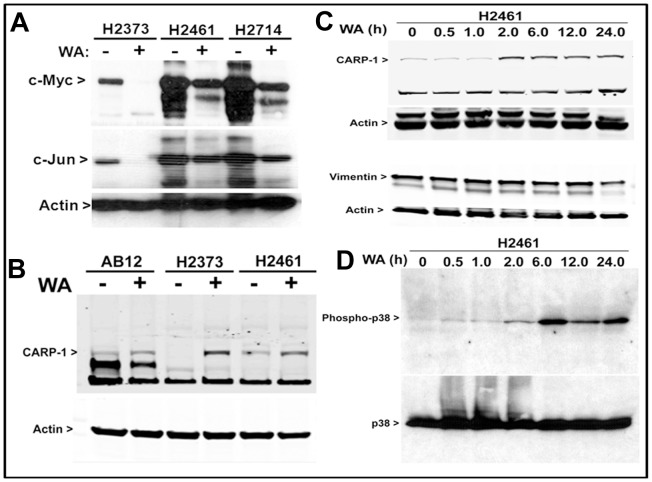
WA suppresses MPM survival and metastasis promoting genes while enhances expression/activation of pro-apoptotic genes. MPM cells were either untreated (DMSO; denoted as −) or treated with 10 µM WA (denoted as +) and cell lysates were analyzed by western blotting as in methods. Levels of c-myc, c-Jun (A), and CARP-1 (B) proteins were determined in the cells treated with WA for 12 h. In addition, MPM cells were either untreated (DMSO, denoted as 0) or treated with 10 µM WA for the indicated time periods, and the levels of CARP-1, vimentin (C), and phospho-p38 (D) were determined by western blotting. The membranes in panels A–C were subsequently analyzed for levels of actin to assess protein loading, while the membrane in panel D was probed for expression of total p38.

The adverse side effects associated with many current anti-cancer therapies together with the molecular complexity of cancers are limiting factors in effective treatment/management of many cancers. For better and improved therapeutic responses it may therefore be necessary to identify additional perhaps novel cancer cell growth inhibitory targets/pathways for potential exploitation in devising efficacious therapeutic strategies. To this end, we utilized gene array-based approaches to further investigate mechanisms of MPM cell growth regulation by WA. H2373 MPM cells were either untreated or separately treated with two doses of WA as described in methods. The RNAs from each group were hybridized with gene-array chips, and the data were computed to identify genes that had a significant 2-fold or higher altered expression following WA treatments. A select subset of these genes is indicated in Table 1 (see table S1 for complete list of WA-regulated genes in H2373 MPM cells). Interestingly, the array data revealed down-regulation of a vast majority of genes while a small number of genes were up-regulated. Among the genes that were down-regulated included cell growth and metastasis-promoting oncogenes c-myc, c-fos, c-jun, while tissue inhibitor of metallopeptidases (TIMP)-2 was significantly upregulated (Table 1). Our western blot analyses further demonstrate that treatments of different human MPM cells with WA resulted in diminished levels of c-myc and c-jun proteins (Fig. 4A).

**Table 1:** List of select WA-regulated genes in H2373 MPM cells.

ID	SYMBOL	Name	ENTREZ	FoldChange	Direction
6380717	HSPA1A	heat shock 70 kDa protein 1A	3303	26.526557	DOWN
5670465	ADM	adrenomedullin	133	25.887796	DOWN
3850433	HSPA1B	heat shock 70 kDa protein 1B	3304	24.628061	DOWN
60079	DNAJB1	DnaJ (Hsp40) homolog, subfamily B, member 1	3337	24.051914	DOWN
870379	ZFAND2A	zinc finger, AN1-type domain 2A	90637	15.772064	DOWN
1340600	PPP1R15A	protein phosphatase 1, regulatory (inhibitor) subunit 15A	23645	11.242614	DOWN
6660601	HMOX1	heme oxygenase (decycling) 1	3162	8.7309379	DOWN
160092	HSPA6	heat shock 70 kDa protein 6 (HSP70B')	3310	8.2488636	DOWN
830619	DDIT3	DNA-damage-inducible transcript 3	1649	7.4905684	DOWN
6860377	DUSP1	dual specificity phosphatase 1	1843	6.8823963	DOWN
4610189	HERPUD1	homocysteine-inducible, endoplasmic reticulum stress-inducible, ubiquitin-like domain member 1	9709	5.7754888	DOWN
5420095	MYC	v-myc myelocytomatosis viral oncogene homolog (avian)	4609	5.2859313	DOWN
150327	HSPH1	heat shock 105 kDa/110 kDa protein 1	10808	5.0773806	DOWN
4390450	SGK1	serum/glucocorticoid regulated kinase 1	6446	5.0676214	DOWN
4880673	GADD45A	growth arrest and DNA-damage-inducible, alpha	1647	4.8698733	DOWN
7570324	ID3	inhibitor of DNA binding 3, dominant negative helix-loop-helix protein	3399	4.6867504	DOWN
870338	EGR1	early growth response 1	1958	4.3381067	DOWN
7160239	FOSB	FBJ murine osteosarcoma viral oncogene homolog B	2354	4.2438425	DOWN
6510367	JUN	jun oncogene	3725	3.972581	DOWN
1710553	HSPA6	heat shock 70 kDa protein 6 (HSP70B')	3310	3.6847097	DOWN
1340075	BAG3	BCL2-associated athanogene 3	9531	3.6837105	DOWN
4210524	RND3	Rho family GTPase 3	390	3.4998418	DOWN
**730286**	**TXNRD1**	**thioredoxin reductase 1**	**7296**	**3.4690154**	**UP**
1260086	ID2	inhibitor of DNA binding 2, dominant negative helix-loop-helix protein	3398	3.2341928	DOWN
4280017	FOS	v-fos FBJ murine osteosarcoma viral oncogene homolog	2353	3.1238524	DOWN
4920110	GADD45B	growth arrest and DNA-damage-inducible, beta	4616	3.0608382	DOWN
4860286	UBB	ubiquitin B	7314	2.7247999	DOWN
670386	ID1	inhibitor of DNA binding 1, dominant negative helix-loop-helix protein	3397	2.7209816	DOWN
**7650358**	**TGFBI**	**transforming growth factor, beta-induced, 68 kDa**	**7045**	**2.3790355**	**UP**
**1170709**	**CDH2**	**cadherin 2, type 1, N-cadherin (neuronal)**	**1000**	**2.3726027**	**UP**
**780270**	**TIMP2**	**TIMP metallopeptidase inhibitor 2**	**7077**	**2.2000615**	**UP**
4040097	KLF6	Kruppel-like factor 6	1316	2.1886976	DOWN
3190148	DDIT4	DNA-damage-inducible transcript 4	54541	2.1817718	DOWN
**830661**	**DCBLD2**	**discoidin, CUB and LCCL domain containing 2**	**131566**	**2.1806644**	**UP**
7050278	RUNX1	runt-related transcription factor 1	861	2.1623177	DOWN
3710544	EIF1	eukaryotic translation initiation factor 1	10209	2.1583103	DOWN
670086	MXD1	MAX dimerization protein 1	4084	2.1341046	DOWN
1470215	MAP3K8	mitogen-activated protein kinase kinase kinase 8	1326	2.1100298	DOWN
3060300	UBC	ubiquitin C	7316	2.0749465	DOWN
6660343	RBM14	RNA binding motif protein 14	10432	2.0674976	DOWN

Genes upregulated by WA are in bold.

CARP-1/CCAR1 is a biphasic transducer of cell growth and apoptosis signaling [19]–[21], [28]. With reference to the MPM model, our previous studies indicated that the proteasome inhibitor velcade as well as the chemo-preventive and dietary agent curcumin suppressed MPM cell growth in part by stimulating CARP-1 expression [5], [29]. Here we investigated whether and to what extent CARP-1 was involved in MPM cell growth inhibition by WA. WA treatments of murine and human MPM cells resulted in elevated levels of CARP-1 (Fig. 4B). H2461 Cells were treated with 10 µM WA for various time periods, and the cell lysates were analyzed by western blotting for CARP-1 expression. WA stimulated CARP-1 levels in a time-dependent manner with robust CARP-1 increase noticeable as early as 2 h of WA treatment (Fig. 4C). A number of studies have described protumorigenic and prometastatic properties of vimentin-expressing cancer cells, and vimentin expression has been found to correlate with poor outcome in many cancers [30]. Although MPM cells are mesothelial in origin and express vimentin, and the fact that WA binds to tetrameric vimentin complex [31], and targets vimentin selectively in the cancer cells [13], we investigated whether WA targeted vimentin in MPM cells. As shown in figure 4C, WA exposure caused reduced levels of vimentin at 24 h of treatment. Since WA promoted apoptosis signaling and caspase 3/7 activation in MPM cells as early as 2 h (see Fig. 3D), and given that activated caspase-3 is known to promote vimetin degradation [13], it is likely that WA-dependent loss of vimentin in MPM cells is in part due to its degradation by caspase-3.

**Figure 5:**
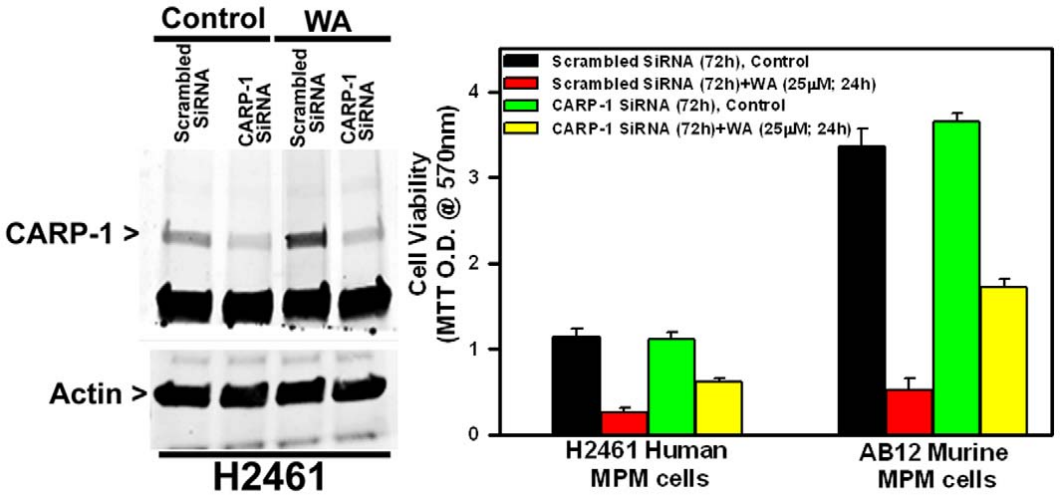
CARP-1 is required for MPM cell growth inhibition by WA. Knockdown of CARP-1 blocks WA effects. Cells were transfected with 100 nM each of scrambled or CARP-1 siRNAs for 72 h and then were either untreated (Control, DMSO) or treated with 10 µM WA for further 24 h. Cell lysates were subjected to western blotting as in Fig. 4 for levels of CARP-1 and actin proteins or subjected to MTT assay for determination of cell viabilities essentially as in Fig. 1. Columns in B represent means of three independent experiments; bars, S.E. * and #, *p* = ,0.01 relative to WA-treated, scrambled siRNA-transfected cells.

Our previous studies have revealed involvement of stress-activated protein kinase (SAPK) p38 in transducing CARP-1-dependent cell growth inhibitory signaling [20], [21]. Because WA treatments stimulated CARP-1 expression in MPM cells (Fig. 4B, C), we next investigated whether WA activated p38, and the extent CARP-1 was required in transducing growth inhibitory effects of WA. Exposure of MPM cells to WA resulted in activation (phosphorylation) of p38 as early as 2 h while robust p38 activation was noticeable at the 6 h and subsequent periods of treatment (Fig. 4D). The extent CARP-1 was required for MPM cell growth inhibition by WA was determined next by experiments involving siRNA-dependent knock-down of CARP-1. H2461 cells were transfected with 100 nM of each of the scrambled or CARP-1 siRNAs essentially as described before [21] for 72 hours and the cells were then either untreated or treated with WA. Western blot analysis of the cell lysates revealed that CARP-1 siRNAs, but not scrambled siRNAs, caused loss of CARP-1 expression (Fig. 5A). Although WA stimulated CARP-1 expression in scrambled siRNA-transfected MPM cells, it failed to increase CARP-1 levels in cells transfected with CARP-1 siRNAs (Fig. 5A). In a separate experiment, human and murine MPM cells were similarly transfected with CARP-1 or scrambled siRNAs followed by their treatment with WA essentially as in Fig. 5A, and cell viabilities were determined by the MTT assay as in methods. WA inhibited growth of MPM cells transfected with scrambled siRNAs, whereas CARP-1 knock-down interfered with WA-dependent suppression of MPM cell growth (Fig. 5B). The data in Figs. 4 and 5 suggest that MPM inhibitory signaling by WA involves down-regulation of oncogenes c-myc and c-jun, activation of pro-apoptotic p38 kinase, and stimulation of CARP-1, while depletion of CARP-1 interferes with WA-dependent MPM cell growth inhibition.

### WA inhibits tumor growth in mesothelioma allograft

**Figure 6:**
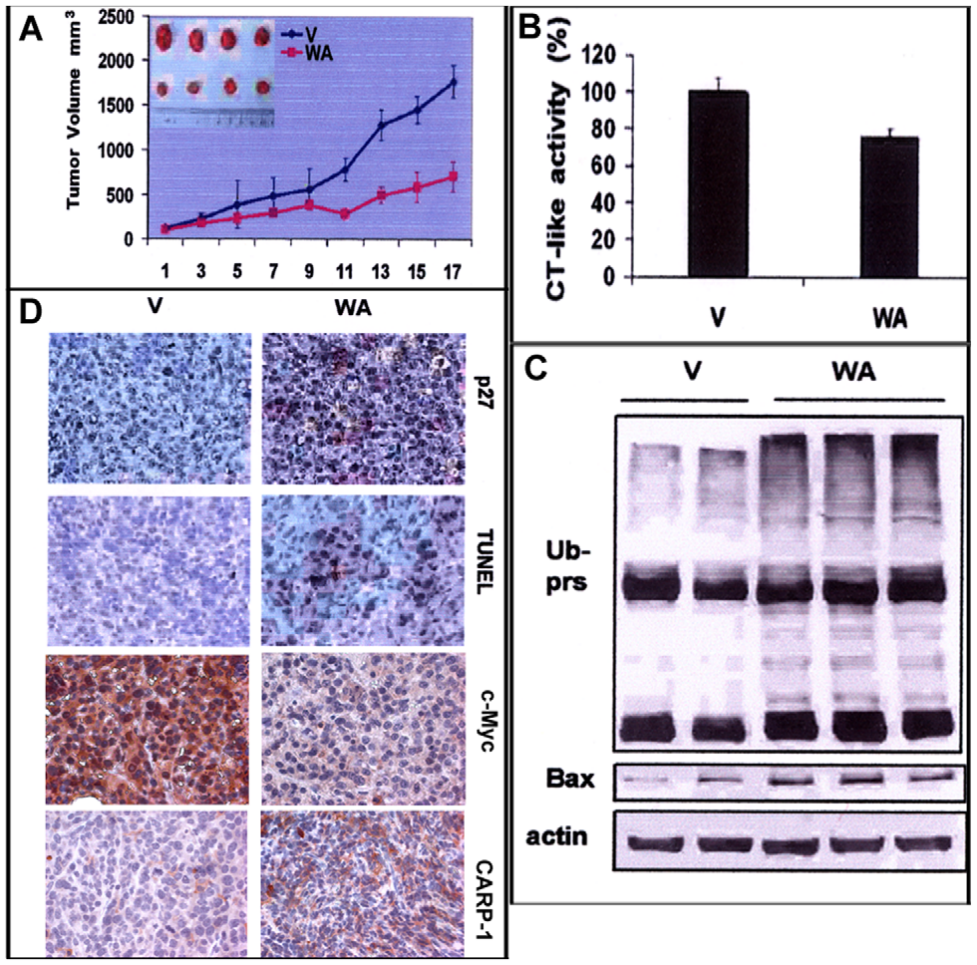
WA inhibited tumor growth in murine MPM allograft. Female BALB/c mice bearing AB 12 tumors were treated with either vehicle (V) or WA as described in methods. Animals were monitored every day for tumor volumes (growth) as well as any activity alteration. Tumor volumes were measured every other day for the control and treated groups (A). *Points*, mean tumor volume in each experimental group containing 4 mice. *Bars*, SD. Tumors were collected after 17 days of treatment, and the tumor biopsies were analyzed by the proteasomal chymotrypsin-like activity assay (*B*) and Western blotting for ubiquitinated proteins (Ub-prs) and Bax (*C*) essentially as detailed in methods. D, The tumor tissues from the control and treated groups were immuno-stained for apoptosis (by TUNEL assay) and levels of CDKI p27, oncogene c-myc and CARP-1 proteins as noted in methods. Magnifications are 400×.

To investigate whether WA inhibits MPM tumor growth, we implanted murine mesothelioma AB12 cells (0.5×10^6^) subcutaneously into the right flanks of female Balb/c mice. When the tumors became palpable (∼120 mm^3^), mice were randomized into the 2 groups for treatment with vehicle control or WA. Doses of 2–5 mg/kg of WA were previously found to be efficacious without significant side effects in animal studies involving xenografts of human breast and prostate cancer cells as well as soft tissue sarcomas (12, 13). Accordingly, the mice bearing AB12 cell-derived tumors were administered with 5 mg of WA/kg of body weight given every day *i.p.* for 17 days. Inhibition of tumor growth by WA was observed after a 17 day–treatment, indicating the efficacy of WA against mesothelioma (Fig. 6A). After 17 days treatment, control tumors grew to an average size of 1774 mm^3^, and WA–treated tumors grew to 714 mm^3^, corresponding to a 60% inhibition (Fig. 6A). Tumor weights were measured by the end of the experiment. The average of tumor weight in vehicle treated group was 1.42 g, while it was 0.46 g in WA-treated group, showing a 68% reduction in tumor weight (data not shown). More importantly, we found absence of any tumor in one mouse in the WA-treated group underscoring a complete response (data not included).

To determine whether WA suppressed murine MPM tumor growth by attenuating proteasome activity *in vivo*, we extracted proteins from the tumor remnants and used them for multiple assays as below. We found that the proteasomal chymotrypsin-like activity was inhibited by 25% in the mesothelioma tumors from mice treated with WA compared to vehicle-treated mice (Fig. 6B). This was associated with the accumulation of ubiquitinated proteins and the proteasome target Bax (Fig. 6C). Increased expression of proteasome targets, p27 was determined following immunostaining of the treated and control tumors using anti-p27 antibodies. Elevated levels of p27 was evident in tumors from mice treated with WA (Fig. 6D), further confirming that WA was able to inhibit tumor proteasome activity *in vivo*. Further, levels of apoptosis in the control and WA-treated tumors were determined using the TUNEL assay, as well as immunostaining the tumors tissues for levels of c-myc and CARP-1 proteins. The TUNEL staining indicated an increase in the number of apoptotic cells in the tumors from mice treated with WA when compared with the corresponding vehicle-treated control (Fig. 6D). Similar to the in vitro studies, c-myc expression was diminished while CARP-1 levels were elevated in tumors derived from mice treated with WA. Taken together, these data show that WA has the ability to regress mesothelioma growth in vivo in part by inhibiting proteasome and stimulating apoptosis.

## Discussion

WA is the major and the most active component of dietary supplement Ashwagandha. Recent preclinical studies have revealed that WA targets multiple molecules for mediating cell death in a variety of cancer cells. WA exhibited anti-angiogenesis effects through NF-κB inhibition, and the ubiquitin-proteasome pathway was thought to be involved [32]. Our previous work has revealed direct inhibition on the proteasomal chymotryptic activity by WA, which likely contributes to cell death induction and tumor growth inhibition in human prostate cancer [14]. WA has also been reported to activate prostate apoptosis response-4 (Par-4) gene, which contributes to regression of PC-3 xenografts in nude mice [12]. WA treatment generated reactive oxygen species (ROS), causing cell death in HL-60 cells [18]. Consistent with these findings, we found that WA treatments increased pro-apoptotic protein Bax and NF-κB inhibitory protein IκB-α in the patient derived MPM cells. WA also decreased the proteasomal chymotryptic activity in human and murine MPM cells, suggesting that WA targets multiple pathways to suppress MPM growth. Cell proliferation and cell death are regulated by the balance between proapoptotic and antiapoptotic proteins [33]. Increased level of pro-apoptotic protein Bax and IκB-α in the presence of WA (Figs. 2, 3) would make the human MPM cells switch to apoptotic cell death. Indeed, WA treatment induced caspase-3 activation, PARP cleavage, and morphological changes (condensed nucleus) characteristic of apoptosis in MPM cells (Figs. 2, 3) strongly underscoring its pro-apoptotic properties.

Simultaneous blocking of cancer cell growth and survival pathways while activating apoptosis is a powerful approach to effectively suppress cancer. Our current studies indicating involvement of apoptosis in WA-dependent inhibition of MPM growth highlights anti-MPM potential for this safe and non-toxic agent. In this context, our studies are consistent with a number of prior studies demonstrating involvement of apoptosis signaling in WA-dependent suppression of human breast and prostate cancer cell growth as well as the soft tissue sarcomas [11]–[13]. The fact that alterations in apoptosis signaling pathways often contribute to MPM growth and survival [34], we undertook an in vitro and gene-array based profiling to identify novel transducers of apoptosis signaling that may be activated/induced by WA. Our studies revealed that while WA suppressed levels of several growth-promoting genes it also caused elevated expression of cell growth inhibitory and apoptosis transducers such as CCAR1/CARP-1, and TIMP2. Our gene-array-based analyses (table 1) together with western blot data in figs. 4 and 5 convincingly reveal a new group of apoptosis signal transducing genes that are activated by WA in MPM cells in vitro and in vivo. Since, CCAR1/CARP-1 is an emerging and novel target of diverse cell growth and apoptosis signaling pathways [19]–[21] and is not involved in cisplatin-dependent HBC growth inhibition [24], identification of CCAR1/CARP-1 as a downstream effector of WA signaling would be useful for design of effective strategies for optimizing WA potential as suppressor of MPM and its drug (Cisplatin)-resistant phenotype.

The gene array data also revealed a number of interesting genes that may be involved in regulating MPM cell growth and survival in the presence of WA. Of note is the down regulation of oncogene c-myc, and AP-1 transcription factor components c-fos and c-jun (table 1). Since c-myc and AP-1 signaling are down-stream of the growth and metastasis-promoting Ras-Raf-Erk pathway, whether and to the extent WA targets upstream regulators of this pathway to block MPM growth and metastases remains to be clarified. WA treatments however resulted in activation of pro-apoptotic p38 MAPK/SAPK that is often known to transduce stress-dependent inhibitory signals. Moreover, WA not only suppressed levels of the tumorigenesis and metastasis-associated vimentin protein, it also caused elevated levels of cancer cell inhibitory, extra cellular matrix-associated TIMP2 protein. Thus the mechanism of action of WA would appear to involve pleiotropic effects including (a) activation of signal transduction pathways, (b) stimulation of apoptosis, (c) blockage of proteasome, and (d) inhibition of growth and metastasis-promoting genes such as c-myc and vimentin.

WA exerted potent inhibition on the growth of mesothelioma AB12 allografts. Consistent with the anti-MPM effects of WA i*n vitro*, inhibition of proteasome also correlated with cell death induction *in vivo*. Treatment of tumors with WA, but not vehicle, caused inhibition of proteasomal chemotrypsin-like activity and accumulation of its substrate Bax as well as ubiquitinated proteins (Fig. 6). The proteasome inhibition also resulted in extensive accumulation of p27 *in situ* in WA-treated tumors (Fig. 6D). The WA-treated tumors also elicited TUNEL positivity and morphological features associated with apoptosis, demonstrating that proteasome inhibition by WA also triggers apoptosis in vivo (Fig. 6D). Finally, since WA treatments caused regression of murine MPM tumor growth (Fig. 6A) to the extent that a complete regression of tumor growth was in noted in one WA-treated animal, suggests that WA can reach a therapeutic concentration *in vivo* to facilitate its direct targeting and inhibition of tumor cellular proteasome, resulting in elevated apoptosis induction and tumor growth inhibition.

In brief, the data presented here convincingly demonstrate that WA targets multiple pathways to suppress MPM growth *in vitro* and *in vivo*, and underscore its potential as a future anti-MPM therapy.

## Supporting Information

Table S1 **List of WA-regulated genes in H2373 MPM cells.**
(XLS)
