# Correction: Withaferin A Inhibits the Proteasome Activity in Mesothelioma *In Vitro* and *In Vivo*

**DOI:** 10.1371/annotation/1f7766a6-35da-4d34-b07b-4c06667bdbec

**Published:** 2013-02-08

**Authors:** Huanjie Yang, Ying Wang, Vino T. Cheryan, Wenjuan Wu, Cindy Qiuzhi Cui, Lisa A. Polin, Harvey I. Pass, Q. Ping Dou, Arun K. Rishi, Anil Wali

The authors would like to make the following addition to the Acknowledgments section of this article: "The authors also wish to thank Dr. Vinesh Thidil Puliyappadamba for performing and providing images for the western blot in Figure 4A."

